# Factors Associated With Changes in Alcohol Use During Pregnancy and the Postpartum Transition Among People With HIV in South Africa and Uganda

**DOI:** 10.1177/23259582231161029

**Published:** 2023-03-22

**Authors:** Amelia M. Stanton, Benjamin D. Hornstein, Nicholas Musinguzi, Brett Dolotina, Catherine Orrell, Gideon Amanyire, Stephen Asiimwe, Anna Cross, Christina Psaros, David Bangsberg, Judith A. Hahn, Jessica E. Haberer, Lynn T. Matthews, For the META Study Team

**Affiliations:** 1 Department of Psychological and Brain Sciences, 1846 Boston University, Boston, USA; 2 446213 The Fenway Institute, Boston, USA; 3 Department of Psychiatry, 2348 Massachusetts General Hospital, Boston, USA; 4 Department of Medicine, 9967 University of Alabama at Birmingham, Birmingham, USA; 5 Makerere-Mbarara Universities Joint AIDS Program (MJAP), Mbarara, Uganda; 6 108123 Mbarara University of Science and Technology, Mbarara, Uganda; 7 Department of Epidemiology, 33638 Mailman School of Public Health, Columbia University, New York, USA; 8 Desmond Tutu Health Foundation, Cape Town, South Africa; 9 Institute of Infectious Disease and Molecular Medicine and Department of Medicine, University of Cape Town, Cape Town, South Africa; 10 560159 Africa Health Research Institute, Durban, South Africa; 11 Global Health Collaborative, Mbarara, Uganda; 12 Kabwohe Clinical Research Center (KCRC), Kabwohe, Uganda; 13 1811 Harvard Medical School, Boston, USA; 14 School of Public Health, Oregon Health and Science University/Portland State, Portland, USA; 15 Department of Medicine, 8785 University of California San Francisco, San Francisco, USA; 16 Center for Global Health, 2348 Massachusetts General Hospital, Boston, USA

**Keywords:** HIV, sub-Saharan Africa, alcohol use, pregnancy

## Abstract

Identifying factors associated with alcohol use changes during pregnancy is important for developing interventions for people with HIV (PWH). Pregnant PWH (n = 202) initiating antiretroviral therapy in Uganda and South Africa completed two assessments, 6 months apart (T1, T2). Categories were derived based on AUDIT-C scores: “no use” (AUDIT-C = 0 at T1 and T2), “new use” (AUDIT-C = 0 at T1, >0 at T2), “quit” (AUDIT-C > 0 at T1, =0 at T2), and “continued use” (AUDIT-C > 0, T1 and T2). Factors associated with these categories were assessed. Most participants had “no use” (68%), followed by “continued use” (12%), “quit” (11%), and “new use” (9%). Cohabitating with a partner was associated with lower relative risk of “continued use.” Borderline significant associations between food insecurity and higher risk of “new use” and between stigma and reduced likelihood of “quitting” also emerged. Alcohol use interventions that address partnership, food security, and stigma could benefit pregnant and postpartum PWH.

## Introduction

Alcohol use disorder (AUD) is prevalent among people with HIV (PWH) in sub-Saharan Africa, with 22% reported as the average 1-year prevalence of AUD in a recent meta-analysis. The prevalence of AUD in PWH differs by country and is higher in South Africa (SA) (29%), for example, than in Uganda (17%).^1^ Alcohol use, heavy episodic drinking, and AUD is especially problematic in the context of HIV due to associations with antiretroviral therapy (ART) non-adherence.^2–6^ In addition to negative effects on ART adherence, alcohol use may directly compromise immunological functioning and viral suppression.^7–9^ Studies have investigated prevalence rates of alcohol use during pregnancy among PWH in sub-Saharan Africa. Rates range by country, but are consistently high in SA and Uganda. In a SA-based sample, 18% of participants reported drinking during pregnancy, and of those individuals, 67% usually binged when drinking (defined as 3 or more drinks in one sitting,^10^ though definitions of binging vary; 4^11,12^ or 5^13^ drinks in one sitting are more commonly used definitions). In a 2016 meta-analysis, 21% of pregnant women in Uganda reported alcohol consumption (of any amount).^11^ More recently, 24% of pregnant women in northern Uganda reported use of any amount of alcohol during pregnancy.^14^

During pregnancy, alcohol use compromises the physical health of the pregnant individual, has deleterious health consequences on the developing fetus, and may increase the likelihood of perinatal HIV transmission. The negative health implications for both the pregnant person and the developing fetus include stillbirth, low birthweight, spontaneous abortion, and preterm birth.^15–17^ For example, infants born to SA women with HIV who reported hazardous drinking were twice as likely to be small-for-gestational age,^18^ which is associated with greater risk for mortality.^19^ Alcohol use during pregnancy also puts the fetus at risk for fetal alcohol spectrum disorders, which are associated with a range of physical defects, congenital anomalies, as well as deficits in cognitive, behavioral, and emotional functioning.^20,21^ The association between low adherence to ART and alcohol use also increases the risk of perinatal HIV transmission during pregnancy and breastfeeding. Moreover, the negative impacts of alcohol use on HIV care engagement are long-lasting. A longitudinal analysis of a cohort recruited in Cape Town during pregnancy and followed for 5 years revealed that alcohol use was significantly related to reduced uptake of HIV care and adherence to ART over time.^22^ These data highlight the importance of addressing alcohol use during pregnancy among PWH and understanding the factors that may drive changes in alcohol use during this period.

Factors associated with alcohol use during pregnancy have been investigated in a systematic review and several country-specific studies, with few reports examining correlates of alcohol use during pregnancy among PWH. Knowledge about and attitudes toward alcohol use during pregnancy,^23,24^ higher income and higher social status (among participants living in high-income countries),^25^ having a partner or friend who drinks,^26^ and higher levels of pre-pregnancy alcohol consumption predict alcohol use during pregnancy. Notably, women with more children and a higher number of previous pregnancies were more likely to drink,^27,28^ whereas women with higher levels of educational attainment were less likely to drink.^28^ Pregnant PWH may have concerns about managing their HIV, transmitting HIV to their fetus or newborn, and the ability to take care of their child in the long term. Alcohol use can be used as a strategy to cope with those HIV-related stressors as well as with stressors unrelated to HIV, including unplanned pregnancies, resistance to parenthood, and the potential loss of social connections during a difficult period of life transition.^29^ Among pregnant PWH in SA, alcohol use during pregnancy has been linked to being single or unpartnered, experiences of intimate partner violence, and lower levels of HIV-related stigma.^30^ In the same sample, hazardous alcohol use was also associated with intimate partner violence.^30^ Another SA-based analysis found that women with poorer mental health, who used tobacco, or had a greater history of engaging in sexual-risk taking behaviors were more likely to drink during pregnancy, whereas married women were less likely to do so.^10^

Equally important and much less known are factors associated with *changes* in alcohol use during pregnancy and postpartum. Understanding the factors that are associated with increases or decreases in alcohol use over the course of pregnancy and/or breastfeeding may inform alcohol use intervention development. Moreover, identifying the individuals who are more likely to continue using alcohol during pregnancy and breastfeeding may direct providers to the subpopulations of PWH who could benefit most from additional services and resources, both of which are limited in most sub-Saharan contexts. Using scarce resources where they are most needed is an important strategy for both enhancing and sustaining behavioral health programs, including alcohol use services, within antenatal care. To that end, we sought to conduct a preliminary investigation of factors associated with changes in alcohol use over a 6-month period among pregnant PWH in SA and Uganda.

## Methods

### Recruitment, Participants, and Procedures

This is a secondary analysis of data from two timepoints of a longitudinal observational study (“Measuring Early Treatment Adherence” [META]; ClinicalTrials.gov, NCT 02419066)^31^ that assessed adherence to ART among PWH who were initiating treatment in Cape Town, SA and southwestern Uganda between March 2015 and October 2017. South African participants were recruited from 4 health centers in Gugulethu, a former township community outside of Cape Town. In Uganda, participants were recruited from 5 different health centers in or near Mbarara, largely rural areas located around 275 km from Kampala. The META study recruited 3 distinct groups of participants: (1) “early ART/not-pregnant,” which included men and non-pregnant women who were initiating ART with asymptomatic, early-stage HIV infection (defined as CD4 > 350 cells/mm^3^); (2) “early ART/pregnant,” which included pregnant women who were initiating ART with asymptomatic, early-stage HIV infection (defined as CD4 > 350 cells/mm^3^); and (3) “late ART/not-pregnant,” which included men and non-pregnant women who were initiating ART with late-stage HIV infection (defined as CD4 < 200 cells/mm^3^).

In the current analysis, we included participants in the “early/pregnant” group to assess for factors associated with changes in alcohol use during pregnancy. Pregnant participants were eligible if they had a gestational age of 34 weeks or less. Other inclusion criteria were as follows: being naïve to ART and initiating within 28 days of study enrollment, aged 18 years or older, living within 60 km of the clinic, and planning to stay in the area for the next year. Participants were excluded if they had cognitive impairment such that they could not communicate in the local language (ie, isiXhosa in SA and Runyankole in Uganda) and/or provide informed consent.

In brief, the META study included 3 assessment time points: baseline, 6 months, and 12 months. To be included in the current analysis, participants were pregnant at either the baseline assessment or the 6-month assessment; this sample allowed for a comparison of self-reported alcohol use at the pregnancy timepoint and the subsequent timepoint. Please see Haberer and colleagues for a detailed description of META study procedures^31^ and Matthews and colleagues for a description of methods specific to pregnancy and pregnant participants.^32^

### Measures

#### Alcohol use

Self-reported alcohol use at each of the 3 assessments was measured via the 3-item Alcohol Use Disorders Identification Test—Consumption (AUDIT-C),^33^ which is scored on a scale from 0 to 12. Lower scores represent less self-reported alcohol intake. The AUDIT-C was modified to assess for alcohol use in the past 3 months (rather than alcohol use over the past year, as is measured by the original scale).

Based on existing literature, 4 categories of factors were assessed for possible association with changes in alcohol use: demographic, psychosocial, structural, and HIV disease:

1. The demographic factors were location (SA vs Uganda), age, relationship status, education level, employment, and gestational age at pregnancy visit, all of which were assessed at the baseline visit.
2. The psychosocial factors were depression, perceived HIV-related stigma, maladaptive coping, and mental well-being. Depression symptoms were measured with the Depression Scale of the Hopkins Symptom Checklist,^34^ which consisted of 12 items that each assessed the degree to which a symptom of depression was experience over the past week. Per reports that somatic symptoms may overestimate depression among PWH,^35–37^ those items were removed. HIV-related stigma was assessed via the perceived negative attitudes toward PWH subscale of the Berger HIV Stigma Scale.^38^ Items that are scored on a 4-point Likert scale, and higher scores reflect greater perceived HIV-related stigma. Maladaptive coping was assessed with an adapted version of the modified Brief COPE that was altered for PWH^39^; the adapted version that was used in the META study includes 7 Likert-style items (eg, I’ve been saying to myself “this isn’t real,” I’ve been giving up trying to deal with it, I’ve been refusing to believe that it has happened) with higher scores indicating maladaptive coping. Finally, overall mental well-being was measured with a 12-item version of the Medical Outcomes Study HIV Health Survey,^40^ which assesses different domains of functioning, including general well-being.
3. Two structural factors were included in the analysis: food insecurity and structural barriers to care engagement. Food insecurity was measured using the Household Food Insecurity Access Scale,^41^ with higher scores indicating more severe food insecurity. Scores can be used to assign households to different categories of food insecurity, including food secure, mildly food insecure, moderately food insecure, and severely food insecure.^41^ Structural barriers to care were assessed with the Structural Barriers to Clinic Attendance scale,^42^ which was developed in SA for PWH. The scale has 12 items that pertain to transport difficulties and patients’ experiences at the clinic (ie, the patient-provider relationship, wait times, overcrowding, fear of being identified as HIV positive).
4. The HIV disease factors were CD4 count and viral load. At each assessment visit, participants provided blood for CD4 count measurements and viral load assessments (determined by Cobas Taqman Test in Uganda and Roche CAP/CTM HIV-1 v2 assay in SA).

### Statistical Analyses

We derived our primary outcome from self-reported alcohol consumption (ie, AUDIT-C scores) at the pregnancy assessment and at the subsequent assessment, which could have either been the 6-month assessment or the 12-month assessment. That is, some participants were pregnant at baseline; for those individuals, the 6-month assessment was their subsequent assessment. For participants who were pregnant at the 6-month assessment, their subsequent assessment was at 12 months. The 4 derived categories that we used in the analyses are as follows:

1. “No use”: Participants who were not consuming alcohol at the pregnancy visit (AUDIT-C = 0) and at the subsequent visit (AUDIT-C = 0).
2. “New use”: Participants who were not consuming alcohol at the pregnancy visit (AUDIT-C = 0) and reported alcohol use at the subsequent visit (AUDIT-C > 0).
3. “Quit”: Participants who were consuming alcohol at the pregnancy visit (AUDIT-C > 0) and reported no use at the subsequent visit (AUDIT-C = 0).
4. “Continued use”: Participants who were consuming alcohol at both the pregnancy visit and at the subsequent visit (AUDIT-C > 0 at both timepoints).

Categorical variables were summarized using percentages and compared using Fisher's exact test, while numeric variables were summarized using median and interquartile range and compared using the Wilcoxson Ranksum test. To assess factors associated with our primary outcome, we first performed univariable multinomial regression models considering the “no use” category as our reference group. Then, we used a multivariable multinomial logistic model to consider relationships between all variables whose univariable *P*-value was less than .1 and the 4 derived alcohol use categories. Based on previous research, stigma based on perceived negative attitudes toward PWH and mental well-being were considered as *a priori* confounders,^30,43^ so they were retained in the multivariable multinomial model. All variables retained in the final multivariable model had univariate *P*-values less than .05, except for the two a priori confounders. Importantly, the alpha level for the final multivariable model was .05.

Results are presented as relative risk reductions (RRR), defined as the difference in event rates between two groups, expressed as a proportion of the event rate in the treated or exposed groups (ie, the “new use,” “quit,” and “continued use groups” in our analyses) compared to the untreated or unexposed group^44^ (ie, the “no use” category). Epidemiologists have suggested that RRR is a more clinically meaningful measure of an effect than an odds ratio (OR), which can be difficult to interpret and only approximate the relative risk in certain restrictive settings.^45^

In addition, to assess factors associated with stopping all alcohol use relative to continuing use, we ran an exploratory sub-analysis that restricted the sample to only participants in the “quit” and “continued use” groups (n = 45), with “continued use” as the reference. By identifying targets and/or resilience factors associated with quitting that could be leveraged in a future alcohol use cessation or reduction intervention, this comparison may have high clinical utility.

All models were performed using the Huber-White robust standard deviation and analysis was conducted in Stata version 15.1.

### Ethical Approval and Informed Consent

This study was approved by all relevant institutional review boards: Mass General Brigham (2014P002620), Mbarara University of Science and Technology (MUIRC1/7), Uganda National Council for Science and Technology (HS 1667), University of Cape Town (797/2014), Western Cape Province, SA (WC_2015RP1_55), and the City of Cape Town (6502). All participants provided written informed consent.

## Results

### Participant Characteristics

The full sample included 202 pregnant PWH, with 133 participants based in southwestern Uganda and 69 participants based in SA. The mean age of the full sample was 26.2 years (SD = 5.3). See Table 1 for a detailed breakdown of the demographic psychosocial, structural, and HIV disease-related factors by country.

**Table 1.** Demographic, Psychosocial, Structural, and HIV Disease-Related Factors by Country.

	Full sample	Uganda	South Africa	*t* / χ² / *z*	*P*-Value
	*N* *=* *202*	*N* *=* *133*	*N* *=* *69*		
Age, *M (SD)*	26.2 (5.3)	26.1 (5.4)	26.4 (5.1)	−0.5	.6
Relationship status, *N (%)*					
Married	106 (52.5)	102 (76.7)	4 (5.8)	108.6	<.001
Widowed	2 (1.0)	1 (0.8)	1 (1.5)		
Cohabitating	16 (7.9)	7 (5.3)	9 (13.0)		
Single	67 (33.2)	14 (10.5)	53 (76.8)		
Divorced/Separated	11 (5.5)	9 (6.8)	2 (2.9)		
Education level, *N (%)*					
None	4 (3.0)	4 (2.0)	0 (0.0)	15.3	.002
P1-P6/G1-G7	39 (19.3)	35 (26.3)	4 (5.8)		
P7-S6/G8-G12	143 (70.8)	84 (63.2)	59 (85.5)		
>S6/>G12	16 (7.9)	10 (7.5)	6 (8.7)		
Employment status, *N (%)*					
Employed	112 (55.4)	93 (69.9)	19 (27.5)	33.0	<.001
Unemployed	90 (44.6)	40 (30.1)	50 (72.5)		
Number of living biological children, *Median (IQR)*	1 (1,2)	2 (1,3)	1 (1,2)	2.5	.012
Gestational age at baseline visit, *Median (IQR)*	20 (13, 26)	19.5 (12, 24)	24 (18, 28)	−4.1	<.001
Participants still pregnant at follow-up visit, *N (%)*	39 (19.31)	37 (27.8)	2 (2.9)		<.001
CD4 count, *Median (IQR)*	555 (429, 675)	573 (454, 691)	477 (408, 675)	2.0	.047
Log viral load, *Median (IQR)*	8.3 (5.3, 10.0)	7.5 (4.0, 9.5)	9.5 (7.5, 10.3)	−4.0	<.001
Viral suppression, *N (%)*	58 (29)	49 (37)	9 (13)	12.5	<.001
Depression, *M (SD)*	1.6 (0.6)	1.5 (0.6)	1.8 (0.6)	−5.0	<.001
HIV-related stigma, *M (SD)*	2.1 (1.9)	2.2 (0.4)	2.3 (0.2)	1.1	.29
Maladaptive coping, *M (SD)*	2.3 (0.3)	2.2 (0.4)	2.3 (0.2)	−1.8	.078
Mental well-being, *M (SD)*	45 (11)	49 (11)	37 (8)	7.6	<.001
Food insecurity score, *M (SD)*	8 (7)	6 (5)	13 (7)	−6.1	<.001
Secure, *N (%)*	45 (22.3)	35 (26.3)	10 (14.5)	33.9	<.001
Mildly insecure, *N (%)*	15 (7.4)	13 (9.8)	2 (2.9)		
Moderately insecure, *N (%)*	63 (31.2)	52 (39.1)	11 (15.9)		
Severely insecure, *N (%)*	79 (39.1)	33 (24.8)	46 (66.6)		
Structural barriers to care engagement, *M (SD)*	6 (8)	2 (5)	15 (7)	**−**10.9	<.001

*Note*. M, SD, and IQR are used to represent mean, standard deviation, and interquartile range, respectively. P1-P6 indicate Primary 1 to Primary 6 in Uganda, and G1-G7 are Grade 1 to Grade 7 in South Africa. P7-S6/G8-G12 represent completion of high school, and >S6 is attendance at university or other tertiary institutions.

With respect to the psychosocial and structural factors that were assessed, participants in Uganda had significantly lower depression scores over the past week than did participants in SA. Ugandan participants also had significantly greater mental well-being, significantly less food insecurity, and significantly fewer structural barriers to care engagement relative to their counterparts in SA (Table 1).

With respect to the HIV disease-related factors, participants in Uganda had significantly higher CD4 counts and were more likely to be virally suppressed than participants in SA, indicating greater disease severity in SA relative to Uganda. Again, see Table 1 for more details.

### Alcohol Use

As described above, we categorized participants based on their self-reported alcohol use during the baseline visit and at the 6-month follow-up visit. The percentages of participants by country within each of the 4 self-reported alcohol use categories are presented in Table 2.

**Table 2.** Alcohol Use Groups by Country of Origin.

	Full sample	Uganda	South Africa	*t* / χ² / *z*	*P*-Value
	*N* *=* *202*	*N* *=* *133*	*N* *=* *69*		
Severity of alcohol use (AUDIT), *M (SD)*					
At baseline visit	0.9 (1.9)	0.6 (1.4)	1.4 (2.6)	−1.9	.07
At follow-up visit	0.7 (1.7)	0.4 (1.0)	1.4 (2.5)	−2.8	.005
Any alcohol use, *N (%)*					
At baseline visit	47 (23.3)	27 (20.3)	20 (29.0)	1.9	.17
At follow-up visit	43 (21.3)	22 (16.5)	21 (30.4)	5.2	.02
AUDIT > 3, *N (%)*					
At baseline visit	22	8	14	9.5	.002
At follow-up visit	18	4	14	16.7	.000
Alcohol use categories, *N (%)*					
“***No use***”: No alcohol use at baseline visit and no alcohol use at follow-up	137 (67.8)	99 (74.4)	38 (55.1)	2.80	.005
“***New use***”: No alcohol use at baseline visit to any alcohol use at follow-up	18 (8.9)	7 (5.3)	11 (15.9)	−2.53	.01
“***Quit***”: Alcohol use at baseline visit to quit/no alcohol use at follow-up	22 (10.9)	12 (9.0)	10 (14.5)	−1.18	.24
“***Continued use***”: Alcohol use at baseline visit to continued use at follow-up	25 (12.3)	15 (11.3)	10 (14.6)	−0.66	.51
Alcohol use categories among participants who were pregnant at both time points, *N (%)*					
	*N* *=* *39*	*N* *=* *37*	*N* *=* *2*	*t* / χ² / z	*P-value*
				0.73	.87
***“No use”***	29 (74.4)	*27 (73.0)*	*2 (100)*		
***“New use”***	1 (2.6)	*1 (2.7)*	*0 (0)*		
***“Quit”***	3 (7.7)	*3 (8.1)*	*0 (0)*		
***“Continued use”***	6 (15.4)	*6 (16.2)*	*0 (0)*		
Alcohol use categories among participants who were postpartum at the 6-month follow-up, *N (%)*					
	*N* *=* *163*	*N* *=* *96*	*N* *=* *67*	*t* / χ² / *z*	*P-value*
				*8.69*	*.034*
***“No use”***	108 (66.3)	*72 (75.0)*	*36 (53.7)*		
***“New use”***	17 (10.4)	*6 (6.2)*	*11 (16.5)*		
***“Quit”***	19 (11.7)	*9 (9.4)_*	*10 (14.9)*		
***“Continued use”***	19 (11.7)	*9 (9.4)*	*10 (14.9)*		

*Note*. *M* and *SD* are used to represent mean and standard deviation, respectively. Statistics were not calculated among participants who were pregnant at both time points due to small sample sizes. Membership in the 4 alcohol use categories did not differ by pregnancy status at the follow-up assessment (χ^2^(3) = 3.3, *P* = .3).

Overall, in the full sample, 67.8% (n = 137) of participants fell within the “no use” category. The next largest alcohol use category was the “continued use,” which described 12.3% (n = 25) of participants; these women reported alcohol use both during the baseline visit and at the 6-month follow-up visit. Almost 11% of the sample (n = 22) was categorized into the “quit” group—which included participants who reported alcohol use during the pregnancy visit but not 6 months later. Finally, a smaller subset of women (8.9%, n = 18) were in the “new use” group, such that they reported no alcohol use at the baseline visit and any alcohol use at 6-month follow-up; significantly fewer women in Uganda were in the “new use” category relative to women in SA (χ² = −2.53, *P* = .01; Table 2). We also examined the counts and associated percentages of participants who were pregnant at both assessments across the alcohol use categories (Table 2) and differences in the severity of alcohol use by alcohol use group (Table 3).

**Table 3.** Severity of Alcohol Use Across Alcohol Use Categories by Timepoint.

Alcohol use Group	N	AUDIT at Baseline Visit, *M (SD)*	AUDIT at Follow-up Visit, *M (SD)*
*“No use”*	137	0 (0)	0 (0)
*“New use”*	18	0 (0)	3.5 (2.1)
*“Quit”*	22	3.3 (1.9)	0 (0)
*“Continued use”*	25	4.16 (2.4)	3.28 (2.4)

There were some differences in the demographic, psychosocial, structural, and HIV disease factors by alcohol use category ((Table 4). Among the 4 alcohol use groups, there were statistically significant differences in relationship status (χ² = 22.6, *P* = .04), employment status (χ² = 8.65, *P* = .03), depression (*t* = 8.44, *P* = .04), food insecurity (χ² = 23.8, *P* = .005), and structural barriers to care engagement (*t* = 10.1, *P* = .02).

**Table 4.** Demographic, Psychosocial, Structural, and HIV Disease-Related Factors by Alcohol Use Group.

	Full sample	“No use”	“New use”	“Quit”	“Continued use”	*t* / χ² / *z*	*P*-value
	*N* *=* *202*	*N* *=* *137*	*N* *=* *18*	*N* *=* *22*	*N* *=* *25*		
Age, *M (SD)*	26.1 (5.3)	26.3 (5.2)	26.1 (5.3)	25.6 (5.8)	26.2 (5.3)	0.55	.91
Relationship status, *N (%)*						22.6	.04
Married	106 (52)	81 (59.1)	7 (38.9)	9 (40.9)	9 (36.0)		
Widowed	2 (1)	2 (1.5)	0 (0)	0 (0)	0 (0)		
Cohabitating	16 (8)	11 (8.0)	0 (0)	4 (18.2)	1 (4)		
Single	67 (33)	37 (27.0)	11 (61.1)	8 (36.4)	11 (44)		
Divorced/Separated	11 (5)	6 (4.4)	0 (0)	1 (4.6)	4 (16)		
Education level, *N (%)*						9.3	.41
None	4 (2)	2 (1.5)	0 (0)	1 (4.6)	1 (4)		
P1-P6/G1-G7	39 (19)	26 (19)	1 (11.1)	4 (18.2)	7 (28)		
P7-S6/G8-G12	143 (71)	96 (70.1)	16 (88.9)	17 (77.3)	14 (56)		
>S6/>G12	16 (8)	13 (9.5)	0 (0)	0 (0)	3 (12)		
Employment status, *N (%)*						8.65	.03
Employed	112 (55)	83 (60.6)	10 (55.6)	6 (27.3)	13 (52)		
Unemployed	90 (45)	54 (39.4)	8 (44.4)	16 (72.7)	12 (48)		
Number of living biological children, *Median (IQR)*	1 (1, 2)	1 (1, 2)	1 (1, 2)	1 (1, 2)	2 (1, 2)	0.83	.84
Gestational age at baseline visit, *Median (IQR)*	20 (13, 26)	20 (14, 24)	22.5 (16, 27)	21 (17, 28)	19 (12, 27)	0.77	.77
Participants still pregnant at follow-up visit, *N (%)*	39 (19)	29 (21)	1 (5.6)	3 (13.6)	6 (24)	3.3	.35
CD4 count, *Median (IQR)*	555 (429, 683)	557 (430, 687)	518 (428, 668)	558 (438, 683)	552 (418, 675)	0.10	.99
Log viral load, *Median (IQR)*	8.3 (5.3, 10.0)	8.3 (4.5, 10.0)	8.7 (7.3, 10.3)	7.0 (5.0, 9.0)	9.5 (6.3, 10.10)	4.87	.18
Viral suppression, *N (%)*	58 (29)	45 (33)	1 (6)	6 (27)	6 (24)	6.2	.10
Depression, *M (SD)*	1.6 (0.6)	1.5 (0.6)	1.6 (0.4)	1.8 (0.6)	1.7 (0.6)	8.44	.04
HIV-related stigma, *M (SD)*	2.1 (1.9)	2.3 (1.9)	1.7 (1.6)	1.5 (1.8)	2.1 (1.9)	4.5	.21
Maladaptive coping, *M (SD)*	2.3 (0.3)	2.2 (0.3)	2.3 (0.3)	2.4 (0.4)	2.3 (0.4)	0.5	.42
Mental well-being, *M (SD)*	45 (11)	46 (11)	41 (10)	42 (12)	44 (12)	5.4	.14
Food insecurity score, *M (SD)*	8.3 (6.8)	7.7 (6.4)	11.1 (6.8)	9.1 (7.8)	8.6 (7.5)	4.4	.22
Food insecurity						23.8	.005
Secure, *N (%)*	45 (22)	30 (21.9)	3 (16.7)	4 (18.2)	8 (32)		
Mildly insecure, *N (%)*	15 (7)	9 (6.6)	1 (5.6)	4 (18.2)	1 (4)		
Moderately insecure, *N (%)*	63 (31)	55 (40.2)	2(11.1)	3 (13.6)	3 (12)		
Severely insecure, *N (%)*	79 (39)	43 (31.4)	12 (66.7)	11 (50.0)	13 (52)		
Structural barriers to care engagement, *M (SD)*	6.4 (8.4)	5.8 (8.4)	10.9 (9.9)	8.2 (7.7)	5.4 (7.1)	10.1	.02

*Note*. M, SD, and IQR are used to represent mean, standard deviation, and interquartile range, respectively. P1-P6 indicate Primary 1 to Primary 6 in Uganda, and G1-G7 are Grade 1 to Grade 7 in South Africa. P7-S6/G8-G12 represent completion of high school, and >S6 is attendance at university or other tertiary institutions.

### Factors Associated With Changes in Alcohol Use

We initially assessed the degree of association between the 4 categories of factors (demographic, psychosocial, structural, and HIV disease) and changes in alcohol use over the 6-month period using univariable multinomial regression models. The following constructs had univariable *P*-values less than .1: severe food insecurity (*P* = .008), country (*P* = .02), and cohabitation with a partner (*P* = .02). These 3 constructs were then included in a multivariable, multinomial model alongside the *a priori* confounders, stigma (perceived negative attitudes) and mental health, to assess their respective relationships with the 4 derived alcohol use categories.

Table 5 shows complete results of the multivariable, multinomial model predicting changes in self-reported alcohol use among pregnant women with HIV. Notably, participants had a lower relative risk of belonging to the “continued use” group, relative to the reference “no use” category if they were cohabitating with a partner (RRR 0.30; 95% CI (0.10, 0.88); *P* = .03). In other words, participants had a 70% reduction in the risk of being in the “continued use” category if they were living with a partner. Participants who reported severe food insecurity had a higher relative risk of belonging to the “new use” category compared to the “no use” reference category, but this did not reach statistical significance (RRR 3.20, 95% CI (0.84, 12.28); *P* = .09). That is, belonging to the severe food insecurity category trended toward a 3.2 times increased risk of initiating alcohol use in the time period between the two assessments. Additionally, each unit increase in self-reported stigma (specifically, in the perceived negative attitudes subscale of the Berger HIV Stigma Scale^38^) trended toward a 23% reduction in the relative risk of belonging to the “quit” category (RRR 0.77; 95% CI (0.57, 1.03); *P* = .08) relative to the reference “no use” category.

**Table 5.** Multivariate Multinomial Regression Analyses Examining the Association Between Changes in Alcohol Use and Relationship Status, Food Insecurity, Stigma, and Mental Health.

	***“New use”^a^:*** No Alcohol Use while Pregnant to Any Alcohol Use at Follow-up		**“Quit”*^a^*:** Alcohol Use while Pregnant to Quit/no Alcohol Use at Follow-up		**“Continued use”*^a^*:** Alcohol Use While Pregnant to Continued Alcohol Use at Follow-up	
	RRR (95% CI)	*P*-Value	RRR (95% CI)	*P*-Value	RRR (95% CI)	*P*-Value
Country						
Uganda	Ref		Ref		Ref	
South Africa	1.75 (0.58, 5.26)	.32	1.55 (0.39, 6.20)	.54	0.66 (0.18, 2.44)	.54
Relationship status						
Single	Ref		Ref		Ref	
Partnered (cohabitating or married)	0.73 (0.32, 1.67)	.46	1.40 (0.36, 5.38)	.14	0.30 (0.10, 0.88)	.028
Severe food insecurity	3.21 (0.84, 12.28)	.089	2.11 (0.78, 5.72)	.14	1.99 (0.75, 5.25)	.17
Stigma (perceived negative attitudes)	0.82 (0.61, 1.09)	.17	0.77 (0.57, 1.03)	.08	0.95 (0.73, 1.22)	.68
Mental Health	0.99 (0.95, 1.04)	.84	0.98 (0.93, 1.04)	.51	1.00 (0.96, 1.05)	.88

^a^ Reference group: No alcohol use at baseline visit and no alcohol use at follow-up, or “no use.”

In the small exploratory sub-analysis that restricted the sample to only participants in the “quit” and “continued use” groups (n = 45), with “continued use” as the reference, participants who were cohabitating with a partner trended toward a higher relative risk of belonging to the “quit” category (RRR 3.20, 95% CI (0.84, 12.28); *P* = .089). All other variables in the exploratory model (ie, the same variables included in Table 4) were not significantly associated with alcohol use category.

## Discussion

This secondary data analysis of self-reported alcohol use among pregnant PWH in rural Uganda and urban SA offers information on factors that may be associated with changes in alcohol use in this population. We highlight 3 factors that may be associated with changes in use during pregnancy and the early postpartum period: (1) cohabitation (ie, living with a romantic partner), (2) food insecurity, and (3) HIV-related stigma. These factors could be relevant to future alcohol use reduction and/or cessation intervention development for pregnant and postpartum PWH in sub-Saharan Africa. Cohabitation was associated with a reduced likelihood of continued versus no drinking during pregnancy and over the next 6 months. Severe food insecurity was associated with initiating alcohol use during the period between the pregnancy time point and the follow-up timepoint, compared to no alcohol use, although this did not reach statistical significance. Finally, there was a trend with respect to stigma or perceived negative attitudes toward PWH; participants who reported higher stigma were less likely to quit using alcohol between the pregnancy timepoint and the subsequent assessment. Given these findings, it will likely be important to include multiple levels of intervention (ie, individual, community-level, and structural) in future alcohol reduction/substance use programming for pregnant PWH. Although the standard of care in both Uganda and SA is to counsel against alcohol use during pregnancy, there are few if any alcohol or other substance use reduction services that are specifically tailored to pregnant people.

Pregnant PWH in sub-Saharan Africa face complex, multilayered stressors, often in the context of unintended pregnancy (unintended pregnancy rates are as high as 87% among individuals in SA diagnosed with HIV during the pregnancy^46^), that may lead to alcohol use. Concerns about HIV transmission,^29^ apprehensions specific to pregnancy^47^ (eg, anticipated relationship changes, major adjustments in lifestyle), and conceptualizations of pregnancy itself as a stressor^29^ (ie, a sense of disappointment or shame for being pregnant) can also exacerbate existing challenges. Pregnant women in SA, specifically, have described alcohol use as a means of coping with these multiple stressors and associated negative emotions,^29^ which often continue and may even increase over the course of pregnancy and into the postpartum period rather than abating over time. Moreover, drinking alcohol to reduce the negative impact of stressors that are not specific to pregnancy has been conceptualized as a multidimensional coping strategy among women in SA.^48^ In our sample, 20% of participants either maintained their use or started drinking between the baseline visit and the follow-up. Similarly, in a convenience sample of pregnant and postpartum women collected at venues that serve alcohol in Cape Town, only two women reported that they stopped drinking altogether when they learned about their pregnancy, while the majority reported that they either increased their consumption or drank as much during pregnancy as they did prior to pregnancy.^29^ Although the percentages of continued use differ across studies, these findings suggest that a sizeable proportion of PWH are willing to report alcohol use throughout pregnancy and the postpartum period; the actual proportions of pregnant PWH who drink alcohol is likely higher.^49^ Notably, there were significant differences in the numbers of pregnant PWH in each alcohol use category by context. More pregnant PWH initiated alcohol use over the 6-month time frame in SA relative to Uganda, while more participants in Uganda reported no alcohol use at both timepoints. These differences may correspond to established social norms around drinking in the Cape Town region, where binge drinking is common, especially among young individuals with lower socioeconomic status.^50,51^ There may also be higher levels of stigma associated with drinking in Uganda relative to SA.^52^ This could contribute to underreporting of alcohol use, which was observed among non-pregnant participants in the Ugandan sample^53^ of the parent study. Contextual differences in urban versus rural settings may also drive differences in alcohol use. Although there are limited data from sub-Saharan Africa that assess urban-rural differences in alcohol use, one study based in SA identified higher odds of problem drinking in urban-dwelling women compared to those living in rural settings.^54^

Continued alcohol use during pregnancy and post-delivery may be driven by factors that are related to but distinct from stress management, including cohabitation with a significant other. Cohabitation with a romantic partner was associated with reduced relative risk of continued alcohol use (ie, alcohol use at both timepoints) relative to no use at both assessments. This finding could reflect the value of social and romantic support networks during pregnancy; indeed, pregnant women who reported having support networks that discouraged alcohol use were less likely to drink while pregnant,^55^ and interventions that incorporate partner or peer support have demonstrated efficacy in reducing alcohol use among women.^56,57^ Emotional support has also been associated with HIV medication adherence in this same cohort; in a longitudinal analysis of ART adherence among pregnant and postpartum women in the META study, poorer adherence was associated with less emotional support.^32^ Though the longitudinal analysis did not identify a relationship between ART adherence and partnership status, partners in close proximity may provide pregnant PWH with different types of support in the home environment, thereby potentially reducing psychological, financial, HIV-related, and pregnancy-specific stressors.

Addressing structural factors could also be critical for alcohol use reduction during pregnancy and breastfeeding. In this sample, our findings suggested the potential relevance of food insecurity for alcohol use interventions: participants with severe food insecurity had higher relative risk for “new use” over the 6-month period. The existing literature documents a relationship between food insecurity and alcohol use among pregnant women in sub-Saharan Africa.^58^ Food insecurity may have other negative effects among PWH; in a US-based sample, food insecurity was associated with suboptimal adherence to ART and unsuppressed viral load.^58^ Although studies assessing the relationship between food insecurity and HIV outcomes in sub-Saharan Africa are limited, associations between lack of food and unsuppressed viral load, for instance, may be stronger in contexts where food insecurity is more prevalent.

Strategies that reduce stigma associated with HIV—at both the individual and societal levels, as well as from HIV care providers—could also be an important component of alcohol use reduction interventions for pregnant and postpartum PWH in sub-Saharan Africa. Given the trend toward an association between increased stigma and reduced likelihood of being in the “quit” group compared to the “no use” group, skills that mitigate intersecting stigmas—which, for pregnant PWH, may include HIV-related stigma,^59^ stigma around unplanned pregnancy^29^ as well as stigma related to substance use in general^60^ and substance use during pregnancy^61–63^—could be highly relevant. Among pregnant South African PWH, increased HIV-related stigma was associated with decreased utilization of social support, and social support was associated with increased ART adherence.^59^ Non-pregnant PWH have reported that they use substances, including alcohol, because their HIV-positive identity makes them feel “subhuman” or worthless.^60^ Stigma in general is viewed as a barrier to engaging in the specific program of care that prevents the transmission of HIV to the fetus during pregnancy and to the infant during breastfeeding, linking stigma indirectly to infant HIV infection.^64^ Interventions that incorporate specific stigma reduction strategies (eg, self-affirmation, self-soothing at the individual level; community introspection, self-reflection at the societal level^65^) and incorporate peers who are also living with HIV as interventionists may be best suited to move the needle on stigma.^66^

This study has several limitations that must be noted. First, any conclusions drawn from this relatively small sample of data that were not initially intended to answer complex questions about alcohol use over time among pregnant PWH must be considered exploratory. It would have been ideal to incorporate biological measures of alcohol use (eg, phosphatidylethanol [PEth]) into these analyses, but unfortunately, such measures were only taken at baseline, which precluded a comparison of alcohol over time (see this analysis^49^ by Raggio and colleagues for an analysis of the PEth data at baseline). Future longitudinal studies that assess alcohol use across the peripartum period should include biological measures in addition to self-report tools at each time point. However, the fact that many pregnant PWH, who receive advice on alcohol use cessation as standard of care in both Uganda and SA, did endorse ongoing use indicates that these self-report data may be specific, albeit likely not very sensitive. Importantly, the small size of the derived alcohol use groups at each timepoint and the small number of participants who were pregnant at follow-up (20%, n = 39) made it difficult to model interactions between pregnancy status at the second assessment and the relevant factors (country, relationship status, food insecurity, stigma, mental health) without overfitting the data. Moreover, the small sample size of participants who were pregnant at both timepoints *and* aware of their pregnancy status (ie, for the entire 3-month look-back window of the AUDIT-C) also precluded an analysis that focused exclusively on changes in alcohol use during pregnancy. Future studies should include repeated assessments of alcohol use during pregnancy (both self-report and biological), with shorter look-back windows, to track these changes over time. Though alcohol use during breastfeeding in the postpartum period is associated with some negative infant health outcomes (eg, early cessation of breastfeeding,^67,68^ disruption of infant feeding,^69^ reduction in sleep time^70,71^), these outcomes are far fewer and less severe than those associated with alcohol use during pregnancy. Even though the World Health Organization recommends 6 months of exclusive breastfeeding for women with HIV,^72^ many do not choose to initiate breastfeeding and/or are still counseled not to do so.^73^ Independent of breastfeeding, alcohol use post-delivery may exacerbate other postpartum stressors and negatively impact ART adherence and retention in HIV care.^74^ There is also a strong association between alcohol use postpartum and maternal depression.^75–77^ As another limitation, the parent study did not use the full version of the modified Brief COPE scale, which precluded analysis that might have revealed important relationships between coping styles assessed by specific subscales (eg, avoidance, alcohol use to cope) and the 4 alcohol use groups. These relationships should be assessed in future work. Finally, we did not assess the use of other substances in addition to alcohol. Doing so would have provided a more thorough understanding of factors associated with changes in use of all substances during pregnancy and the postpartum period.

Overall, this analysis offers initial insights on 3 factors that may drive changes in alcohol use during pregnancy and into the postpartum period: social support gained through living with a partner, food insecurity, and HIV-related stigma. These analyses also suggest that traditional, individual-level interventions which convey knowledge about the dangers of alcohol use during pregnancy and are delivered exclusively in clinics or other healthcare settings may not adequately address the contextual factors that lead PWH to sustain or initiate alcohol use during pregnancy and postpartum. Rather, interventions that integrate (1) partner support or some dyadic components (eg, alcohol reduction support groups for couples, couples’ counseling to bolster communication and problem solving around alcohol),^1,2^ (2) income generation solutions, delivery of food parcels, and/or involvement with food cooperatives; and (3) peer- or community-based programming that challenges stigmatizing societal narratives around PWH and alcohol use during pregnancy may best serve this population. Future research should explore the feasibility, acceptability, and efficacy of alcohol use reduction interventions that incorporate these elements.
